# Interferon-Induced Transmembrane Protein 3 Inhibits Hantaan Virus Infection, and Its Single Nucleotide Polymorphism rs12252 Influences the Severity of Hemorrhagic Fever with Renal Syndrome

**DOI:** 10.3389/fimmu.2016.00535

**Published:** 2017-01-03

**Authors:** Zheng Xu-yang, Bian Pei-yu, Ye Chuan-tao, Ye Wei, Ma Hong-wei, Tang Kang, Zhang Chun-mei, Lei Ying-feng, Wei Xin, Wang Ping-zhong, Huang Chang-xing, Bai Xue-fan, Zhang Ying, Jia Zhan-sheng

**Affiliations:** ^1^Department of Infectious Diseases, Tangdu Hospital, Fourth Military Medical University, Xi’an, China; ^2^Department of Microbiology, Fourth Military Medical University, Xi’an, China; ^3^Department of Immunology, Fourth Military Medical University, Xi’an, China

**Keywords:** Hantaan virus, hemorrhagic fever with renal syndrome, interferon-induced transmembrane protein 3, single nucleotide polymorphisms, negative regulator of interferon response

## Abstract

Hantaan virus (HTNV) causes hemorrhagic fever with renal syndrome (HFRS). Previous studies have identified interferon-induced transmembrane proteins (IFITMs) as an interferon-stimulated gene family. However, the role of IFITMs in HTNV infection is unclear. In this study, we observed that IFITM3 single nucleotide polymorphisms (SNP) rs12252 C allele and CC genotype associated with the disease severity and HTNV load in the plasma of HFRS patients. *In vitro* experiments showed that the truncated protein produced by the rs12252 C allele exhibited an impaired anti-HTNV activity. We also proved that IFITM3 was able to inhibit HTNV infection in both HUVEC and A549 cells by overexpression and RNAi assays, likely *via* a mechanism of inhibiting virus entry demonstrated by binding and entry assay. Localization of IFITM3 in late endosomes was also observed. In addition, we demonstrated that the transcription of IFITM3 is negatively regulated by an lncRNA negative regulator of interferon response (NRIR). Taken together, we conclude that IFITM3, negatively regulated by NRIR, inhibits HTNV infection, and its SNP rs12252 correlates with the plasma HTNV load and the disease severity of patients with HFRS.

## Introduction

Hantaan virus (HTNV), the prototype of Hantavirus and harbored mainly by *Apodemus*, is a pathogen that causes hemorrhagic fever with renal syndrome (HFRS) worldwide. Approximately, 150,000 cases of HFRS are reported annually (1). The major clinical characteristics of patients with HFRS include fever, hemorrhage, hypotension, and renal injury (1). China is one of the most severely affected endemic countries of HFRS in the world, with a higher morbidity and mortality rate and more severe manifestations. It has been reported that the plasma HTNV load in the early stage of HFRS associates with the severity of disease, indicating the importance of viremia in the pathogenesis of HFRS (2). Therefore, further studies of host factors limiting HTNV infection and influencing antiviral response as well as disease progression are clinically significant and timely.

The human family of interferon-induced transmembrane proteins (IFITMs) was discovered 25 years ago to consist of interferon-stimulated genes (ISGs) (3). This family includes five members, namely, IFITM1, 2, 3, 5, and 10, among which IFITM1, 2, and 3 possess antiviral activity (4). Different IFITM proteins have different antiviral spectrum (5). For example, IFITM3 has been shown to prevent influenza virus infection *in vitro* and in mice (6, 7), and it also inhibits multiple viruses, including *filoviruses, rhabdoviruses, flaviviruses*, and even Ebola and Zika virus (7–11). The antiviral mechanism of IFITM3 is thought to be the restriction of viral entry into cells (4, 12). Single nucleotide polymorphisms (SNPs) are single nucleotide variations in a genetic sequence that occur at an appreciable frequency in the population. Several SNPs has been identified in IFITM3, among which the rs12252 site with C allele results in a N-terminal truncation of IFITM3 protein, leading to impaired inhibition of influenza virus *in vitro* (13, 14). Notably, the frequencies of rs12252 C allele and CC genotype correlate with disease severity in patients infected with influenza virus (13, 15). HTNV has been shown to induce a type I interferon response (though in later time postinfection) (16, 17). While overexpression of IFITM1, 2, and 3 in Vero E6 cells has been reported to inhibit HTNV infection (18), however, the effect of IFITMs on HTNV infection in human cell lines and its role in HFRS still remain unknown.

LncRNA comprises a group of non-coding RNAs longer than 200 nt that function as gene regulators. Some lncRNAs have been shown to play a role in innate immunity (19). Among them, negative regulator of interferon response (NRIR) (lncRNA NRIR, also known as lncRNA-CMPK2) is a non-coding ISG that negatively regulates IFITM1 and Mx1 expression in HCV infection (20). Notably, IFITM3 is largely homologous to IFITM1, but the role of NRIR in the regulation of IFITM3 in HTNV infection remains unclear.

In the present study, we investigate the effect of IFTTM3 on the replication of HTNV and its role in the development of HFRS in humans. We provide primary evidence suggesting that IFITM3, regulated by NRIR, can inhibit HTNV infection and its SNP rs12252 correlates with the disease severity and viral load in patients with HFRS. This study expands our understanding of the antiviral activity of IFITM3 and enriches our knowledge of innate immune responses to HTNV infection.

## Materials and Methods

### Ethics Statement

This study was conducted in accordance with the recommendations of the biomedical research guidelines involving human participants established by the National Health and Family Planning Commission of China. The Institutional Ethics Committee of Tangdu Hospital approved this study. All subjects gave written informed consent in accordance with the Declaration of Helsinki. Before inclusion, all participants were informed of the study objectives and signed the consent form before blood samples and medical records were obtained.

### Study Participants

Sixty-nine HFRS patients admitted into the Department of Infectious Diseases, Tangdu Hospital between October 2014 and March 2016 were enrolled in this study. All patients were Han Chinese. The diagnosis of HFRS was made based on typical symptoms and signs as well as positive IgM and IgG antibodies against HTNV in the serum assessed by enzyme linked immunosorbent assay (ELISA) in our department. The classification of HFRS severity and the exclusion criteria were described as follows (21): white blood cells (WBC), platelets (PLT), blood urea nitrogen (BUN), serum creatinine (Scr), and heteromorphic lymphocytes that were tested by the Department of Clinical Laboratory (shown in Table 1).

**Table 1:** **Clinical and laboratory characteristics of the study population**.

	Mild patients	Severe patients	*P*-value
Age (years)	45.21 ± 2.87	45.02 ± 2.27	0.9584
Gender (female/male)	6/22	6/35	0.5274
WBC (×10^9^)	16.81 ± 1.82	22.26 ± 1.65	0.0326*
MONO %	12.92 ± 0.97	10.20 ± 0.65	0.0188*
Heteromorphic lymphocytes %	11.32 ± 1.47	10.93 ± 1.42	0.8568
PLT (×10^9^)	64.75 ± 10.59	36.88 ± 5.63	0.0143*
BUN, mmol/l	14.84 ± 1.39	22.69 ± 1.50	0.0005**
Scr, μmol/l	241.30 ± 25.22	407.40 ± 34.14	0.0006**

*WBC, white blood cells; PLT, platelets; MONO %, monocyte percentage; BUN, blood urea nitrogen; Scr, serum creatinine*.

*Values are presented as mean ± SEM*.

P-value refers to the difference between mild and severe patients calculated by unpaired t-test. *P < 0.05, **P < 0.01 compared with mild patients.

According to clinical symptoms and signs, such as fever, effusion, hemorrhage, edema, and renal function, the severity of HFRS can be classified as previously described (21): (1) mild patients were identified with mild renal failure without an obvious oliguric stage; (2) moderate patients were those with obvious symptoms of uremia, effusion (bulbar conjunctiva), hemorrhage (skin and mucous membrane), and renal failure with a typical oliguric stage; (3) severe patients had severe uremia, effusion (bulbar conjunctiva and either peritoneum or pleura), hemorrhage (skin and mucous membrane), and renal failure with oliguria (urine output, 50–500 ml/day) for ≤5 days or anuria (urine output, <50 ml/day) for ≤2 days; and (4) critical patients exhibited ≥1 of the following signs during the illness: refractory shock, visceral hemorrhage, heart failure, pulmonary edema, brain edema, severe secondary infection, and severe renal failure with oliguria (urine output, 50–500 ml/day) for >5 days, anuria (urine output, <50 ml/day) for >2 days, or a BUN level of >42.84 mmol/l. Due to the sample quantity required for SNP typing, the mild and moderate patients were assessed together in the mild group, and we combined severe and critical patients as severe group.

The exclusion criteria for this study were patients with: (1) any other kidney disease, (2) diabetes mellitus, (3) autoimmune disease, (4) hematological disease, (5) cardiovascular disease, (6) viral hepatitis (types A, B, C, D, or E), or (7) any other liver disease. In addition, no patients received corticosteroids or other immunomodulatory drugs during the study period (21).

### Sequencing and Genotyping of SNPs

Genomic DNA was extracted from the peripheral blood of patients using the PureGene DNA Isolation kit (Gentra Systems, Minneapolis, MN, USA). The region encompassing the human IFITM3 rs12252 were amplified by PCR (forward primer, 5′-GGAAACTGTTGAGAAACCGAA-3′ and reverse primer, 5′-CATACGCACCTTCACGGAGT-3′). The PCR products were purified and sequenced using an Applied Biosystems 3730xl DNA Analyzer (Thermo Scientific, Waltham, MA, USA). The allele frequencies and genotypes of healthy Han Chinese and other groups were obtained from the 1,000 genomes project (http://www.1000genomes.org).

### Determination of the Plasma HTNV Load

The HTNV load in plasma samples (collected during the acute phase) from 24 age- and sex-matched HFRS patients with different genotypes were measured using previously reported methods (2). Briefly, viral RNA was extracted from the plasma of HFRS patients using Purelink Viral RNA/DNA Kits (Invitrogen, Carlsbad, CA, USA). The SuperScript III Platinum One-Step Quantitative RT-PCR System kit (Invitrogen, Carlsbad, CA, USA) was employed for the real-time RT-PCR assay. The primers and probe (provided by Sangon Biotech, Shanghai, China) were as follows: forward, 5′-TACAGAGGGAAATCAATGCC-3′, reverse, 5′-TGTTCAACTCATCTGGATCCTT-3′, and probe, 5′-(FAM) ATCCCTCACCTTCTGCCTGGCTATC (TAMRA)-3′. The synthetic S segment of the HTNV standard strain 76–118 RNA transcript was used as the quantitative calibrator. The external standard was the culture supernatant of Vero E6 cells infected with HTNV 76–118, which was quantified using synthetic quantitative calibrators. For each experiment, one aliquot of calibrated 76–118 standard was extracted in parallel with the clinical samples and serially 10-fold diluted with concentrations ranging from 10.56 to 2.56 log_10_ copies/ml. PCR was performed using an iQ5 Cycler (Bio-Rad, Hercules, CA, USA) with following conditions: 42°C for 15 min, 95°C for 2 min, and 50 cycles of 15 s at 95°C, 30 s at 53°C, and 30 s at 72°C. Fluorescence was read during the 72°C step of the final segment of every cycling program.

### Cells, Virus, and IFN-α2a

HUVEC cells (ScienCell Research Laboratories, Carlsbad, CA, USA) were grown in ECM BulletKit (ScienCell Research Laboratories, Carlsbad, CA, USA) in a 5% CO_2_ incubator. A549 cells (ATCC Cat# CRM-CCL-185, RRID:CVCL_0023) were grown in our laboratory in DMEM with 10% FBS (Thermo Scientific, Waltham, MA, USA) in a 5% CO_2_ incubator. Cells were used within passage 10 after primary culture. HTNV strain 76–118 was cultured in Vero E6 cells (ATCC Cat# CRL-1586, RRID:CVCL_0574) in our laboratory and titrated using an immunofluorescence staining assay for HTNV nucleocapsid protein (NP) as previously described (22). The TCID50 was 10^5^/ml, which was calculated using the Reed-Muench method. The recombinant human IFN-α2a was obtained from PBL Interferon Source (Piscataway, NJ, USA) and dissolved in the buffer provided by the manufacturer (composition not disclosed). HUVEC and A549 cells were infected by incubation with HTNV as indicated moi at 37°C for 60 mins. Subsequently, the virus solution was removed and fresh medium added to the cell culture.

### Expression and RNAi of IFITM Molecules

Cells were transfected with lentiviral vectors of c-myc-tagged IFITM1, IFITM2, IFITM3, and IFITM3 NΔ21 (purchased from GENECHEM, Shanghai, China) at a moi of 10. Puromycin (2 μg/ml for HUVEC and 6 μg/ml for A549 cells) was used to create cell lines stably expressing IFITMs. Cells were transfected with control (scrambled) short interfering RNA (siRNA), IFITM1 siRNA, IFITM2 siRNA, or IFITM3 siRNA (10 nM) using Lipofectamine 3000 transfection reagent (Invitrogen, Carlsbad, CA, USA). SiRNAs were purchased from Origene (Rockville, MD, USA), and the sequences were not disclosed.

### Quantitative Real-time PCR Analysis

Total RNA was extracted using TRIzol reagent (Invitrogen, Carlsbad, CA, USA), and cDNA was synthesized using the K1622 kit (Thermo Scientific, Waltham, MA, USA). Quantitative real-time PCR (qPCR) was performed using SYBR Premix Ex Taq II (Takara Biotechnology Co., Dalian, China) with a Bio-Rad iQ5 cycler (Bio-Rad, Hercules, CA, USA). β-actin was used as the reference gene. The primers (Sangon Biotech, Shanghai, China) were as follows:

IFITM1 (forward, 5′-ACTCCGTGAAGTCTAGGGACA-3′ and reverse, 5′-TGTCACAGAGCCGAATACCAG-3′);
IFITM2 (forward, 5′-ATCCCGGTAACCCGATCAC-3′ and reverse, 5′-CTTCCTGTCCCTAGACTTCAC-3′);
IFITM3 (forward, 5′-GGTCTTCGCTGGACACCAT-3′ and reverse, 5′-TGTCCCTAGACTTCACGGAGTA-3′);
IFITM3 pre-mRNA (forward, 5′-CATAGCACGCGGCTCTCAG-3′ and reverse, 5′-CGTCGCCAACCATCTTCCTG-3′);
HTNV S segment (forward, 5′-GCCTGGAGACCATCTGAAAG-3′ and reverse, 5′-AGTATCGGGACGACAAAGGA-3′);
β-actin (forward, 5′-GCTACGTCGCCCTGGACTTC-3′ and reverse, 5′-GTCATAGTCCGCCTAGAAGC-3′);
NRIR (forward, 5′-ATGGTTTTCTGGTGCCTTG-3′ and reverse, 5′-GGAGGTTAGAGGTGTCTGCTG-3′);
NRAV (forward, 5′-TCACTACTGCCCCAGGATCA-3′ and reverse, 5′-GGTGGTCACAGGACTCATGG-3′).

For detection of miR-130a, cDNA was synthesized using the TaqMan microRNA reverse transcription kit (Invitrogen, Carlsbad, CA, USA) with a specific primer in gene-specific TaqMan assay kit (000454, Invitrogen, Carlsbad, CA, USA). MiR-130a level was determined using the gene-specific TaqMan assay kit (000454, Invitrogen, Carlsbad, CA, USA). U6 (001973, Invitrogen, Carlsbad, CA, USA) was used as an endogenous control (23). Because the pre-mRNA levels can represent the initial transcription rate (24), the primers used to detect the pre-mRNA of IFITM3 were designed targeting the intron of IFITM3 as previously described (25). IFITM3 has two exons and one intron. For qPCR of IFITM3 pre-mRNA, the forward primers were positioned in the intron, and the reverse primer was positioned at the beginning of the second exon. For qPCR of IFITM3 mRNA, the forward primers were positioned in the first exon, and the reverse primer was positioned at the beginning of the second exon (24). Because the basal expression of IFITM3 is low in A549 cells, we detected IFITM3 mRNA and pre-mRNA in A549 cells following IFN-α2a treatment (20 IU/ml for 12 h) after the overexpression of NRIR.

### Western Blot Analysis

Cell lysates were prepared using Radio Immunoprecipitation Assay (RIPA) buffer (Sigma-Aldrich, St. Louis, MO, USA). Equal amounts of protein (20 μg protein/lane) were electrophoresed on a 10%-SDS-polyacrylamide gel and electrophoretically transferred to a polyvinylidene difluoride membrane (Millipore, Billerica, MA, USA). After blocking with 5% bovine serum albumin in Tris-buffered saline at room temperature for 1 h, the membranes were incubated with antibodies against IFITM1 (Proteintech Group Cat# 60074-1-Ig Lot# RRID:AB_2233405), IFITM2, IFITM3 (Proteintech Group Cat# 66081-1-Ig Lot# RRID:AB_11182821), and β-actin (Proteintech, Wuhan, Hubei, China) or HTNV NP (provided by the Department of Microbiology, The Fourth Military Medical University) overnight at 4°C. The membranes were then washed and incubated with HRP-conjugated IgG antibody (Cell Signaling Technology, Danvers, MA, USA) for 1 h at room temperature. The blots were developed using an enhanced chemiluminescence detection kit (Millipore, Billerica, MA, USA) and visualized using X-ray film. The blot densities were analyzed using the Quantity One software (Bio-Rad, Hercules, CA, USA). In addition, the RIPA buffer contains 50mM Tris (pH = 7.4), 150 mM NaCl, 1% Triton X-100, 1% sodium deoxycholate, 0.1% SDS. Protease inhibitor cocktail (Roche, Basel, Switzerland) was added before use.

### Immunofluorescence and Confocal Microscopy

The cells were cultured on glass coverslips (Millipore, Billerica, MA, USA) until they were semi-confluence and then incubated with HTNV for 60 min (moi = 1). At the indicated times post-HTNV infection, the cells were fixed with 4% PFA, incubated with 0.3% Triton X-100 (Sigma-Aldrich, St. Louis, MO, USA), and blocked with 5% BSA for 1 h. Following incubation with a mouse monoclonal antibody against c-myc-tag (Sigma-Aldrich, St. Louis, MO, USA, Sigma-Aldrich Cat# M5546), IFITM3, lysosome-associated membrane glycoprotein 1 (LAMP1, Cell Signaling Technology, Danvers, MA, USA), or HTNV NP at 37°C for 2 h, the cells were washed and incubated with anti-rabbit Ig conjugated to Alexa 555 and anti-mouse Ig conjugated to Alexa 488 (Abcam, Cambridge, MA, USA) secondary antibodies at room temperature for 1 h. The nuclei were counterstained with DAPI. An Olympus BX51 fluorescence microscope system and FV1000 confocal microscopy system (Olympus, Tokyo, Japan) were used to capture the images.

### HTNV Binding and Entry Assay

Cells transduced with IFITM3 or the empty vector were detached and washed extensively with cold PBS. The cells and HTNV were pre-chilled on ice for 30 min, mixed at a moi of 1 and incubated at 4°C for 1 h with rotation. Part of cells were washed extensively with ice-cold PBS and harvested for binding assay. Another part of cells were switched to 37°C for 2 h to allow HTNV entry. The HTNV that remained on the cell surface was removed by treatment with proteinase K (0.1 mg/ml, Thermo Scientific, Waltham, MA, USA). To achieve direct entry of HTNV into cells by virus–plasma membrane fusion as a positive control, cells were pre-chilled on ice for 10 min with 20 mM NH_4_Cl. Adsorption of HTNV (moi = 1) was performed at 4°C for 1 h. The cells were then washed, and fusion of the virus with the plasma membrane was triggered by incubation in low pH medium (20 mM sodium succinate, pH = 5.5) for 10 min at 37°C. Infection was followed by incubation for 2 h at 37°C in the presence of 20 mM NH_4_Cl (26). qPCR analysis of the HTNV S segment was conducted to evaluate the influence of IFITMs on HTNV cell adhesion and HTNV entry.

### Statistical Analysis

All data were expressed as the mean ± SEM. Statistical analyses were performed using GraphPad Prism 5 (GraphPad Software, La Jolla, CA, USA). For association analysis of the rs12252 allele and genotype, Fisher’s exact test was used. Independent samples *t*-tests were used for normally distributed data. Differences among groups were determined by one-way analysis of variance (ANOVA) with repeated measures, followed by Bonferroni’s *post hoc* test. *P* < 0.05 was considered statistically significant.

## Results

### The IFITM3 SNP rs12252 C Allele and CC Genotype Associated with Severe HFRS Disease and a Higher Plasma HTNV Load

**Figure 1:**
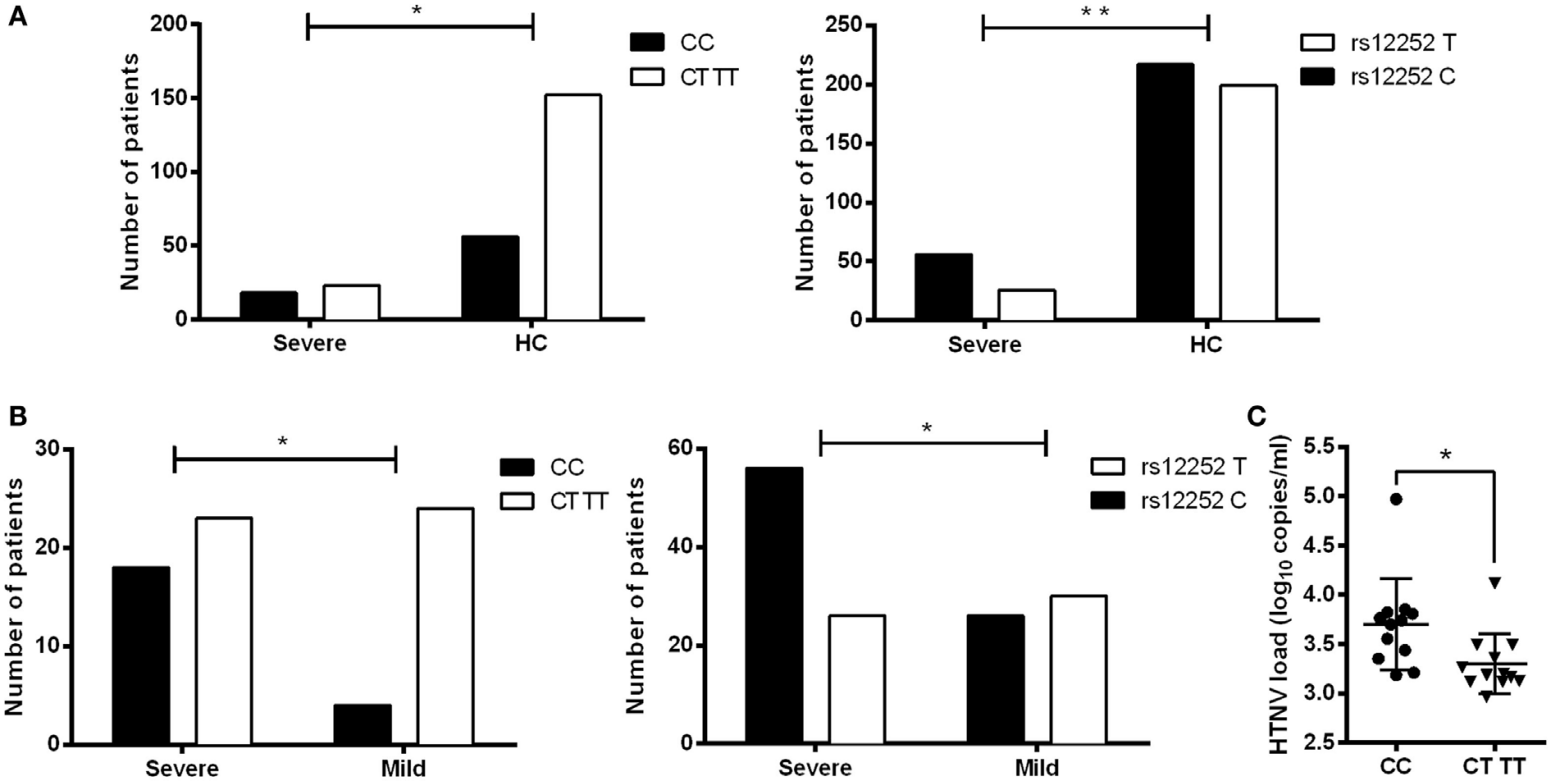
**Association of IFITM3 SNP rs12252C allele and CC genotype with the disease severity and plasma HTNV load in HFRS patients**. **(A)** SNP analysis of rs12252 allele and genotype frequencies between severe HFRS patients and healthy Han Chinese. **(B)** SNP analysis of rs12252 allele and genotype frequencies between severe HFRS patients and mild HFRS patients. Fisher’s exact test was used to test the association between rs12252 allele/genotype and HFRS severity. **(C)** The plasma HTNV load in CC genotype patients and CT/TT genotype patients, tested by qRCR analysis. Each symbol represents one individual patient. Independent samples *t*-test was used to test the difference of HTNV load between groups. *P < 0.05, **P < 0.01.

To determine the clinical significance of IFITM3 SNP in HTNV infection, the relationship between rs12252 SNP and the severity of HFRS in 69 patients were examined. We sequenced 300 bp of the IFITM3 locus encompassing SNP rs12252 in all enrolled patients. Then, we stratified these patients into mild and severe, based on the clinical assessment as described in Section “Material and Methods.” We found a significantly higher frequency of the C allele among severe HFRS patients compared with the healthy Han Chinese in the 1,000 genomes sequence database (68.29 vs. 52.16%, *P* = 0.0076). The frequency of rs12252 C in severe patients was also higher than those mild patients (68.29 vs. 46.43%, *P* = 0.013, Figures 1A,B; Table 2). These data suggest that harboring rs12252 C allele increases the risk of suffering severe disease in HTNV-infected individuals, with an odds ratio (95% CI) of 2.124 (1.067–4.230). For genotypes, 43.90% of the severe patients carried the CC genotype, a significantly higher frequency than the control Han Chinese per 1,000 genomes sequence database (26.92% CC genotype, *P* = 0.03) as well as mildly infected patients (14.29%, *P* = 0.02, Figures 1A,B; Table 2). However, mildly ill individuals did not exhibit a significantly different genotype frequencies compared with the Han Chinese population. In addition, we also found that patients with CC genotype had higher plasma viral load in acute phase (Figure 1C). These results support the notion that the normal function of IFITM3 plays a critical role in the immune response to HTNV infection *in vivo*, which has a substantial influence on the clinical manifestation of HFRS.

**Table 2:** **Allele and genotype frequencies of IFITM3 SNP rs12252 in Chinese HFRS patients and different healthy subjects from the 1,000 genome project**.

	Genotypes from the 1,000 genomes project			HFRS patients from our hospital		
	Northern Europe	European American	Han Chinese	All hospitalized	Mild patients	Severe patients
**Genotype**						
CC	0 (0%)	5 (0.12%)	56 (26.92%)	22 (31.88%)	4 (14.29%)	18 (43.90%)
CT	7 (8.05%)	323 (7.79%)	105 (50.48%)	38 (55.07%)	18 (64.29%)	20 (48.78%)
TT	80 (91.95%)	3816 (92.08%)	47 (22.60%)	9 (13.04%)	6 (21.43%)	3 (7.32%)
Allele C	7 (4.02%)	333 (4.02%)	217 (52.16%)	82 (59.42%)	26 (46.43%)	56 (68.29%)
Allele T	167 (95.98%)	7955 (95.98%)	199 (47.84%)	56 (40.58%)	30 (53.57%)	26 (31.71%)
Statistical analysis*						
Genotype						
*P*-value				0.44	0.17	0.03*
Odds ratio (95% CI)				1.271 (0.7029–2.296)	0.4524 (0.1503–1.362)	**2.124 (1.067–4.230)**
HWE *P*-value				0.24	0.12	0.42
Allele						
*P*-value				0.14	0.48	0.0076*
Odds ratio (95% CI)				1.343 (0.9087–1.984)	0.7948 (0.4542–1.391)	**1.975 (1.194–3.268)**

The Hardy–Weinberg equilibrium (HWE) test was conducted in each patient group. Fisher’s exact test was used for association testing of the rs12252 allele and genotype. *P < 0.05 compared with the Han Chinese. The bold font highlights significant difference.

### IFITM3 SNP rs12252 Polymorphisms Influenced Its Anti-HTNV Activity

**Figure 2:**
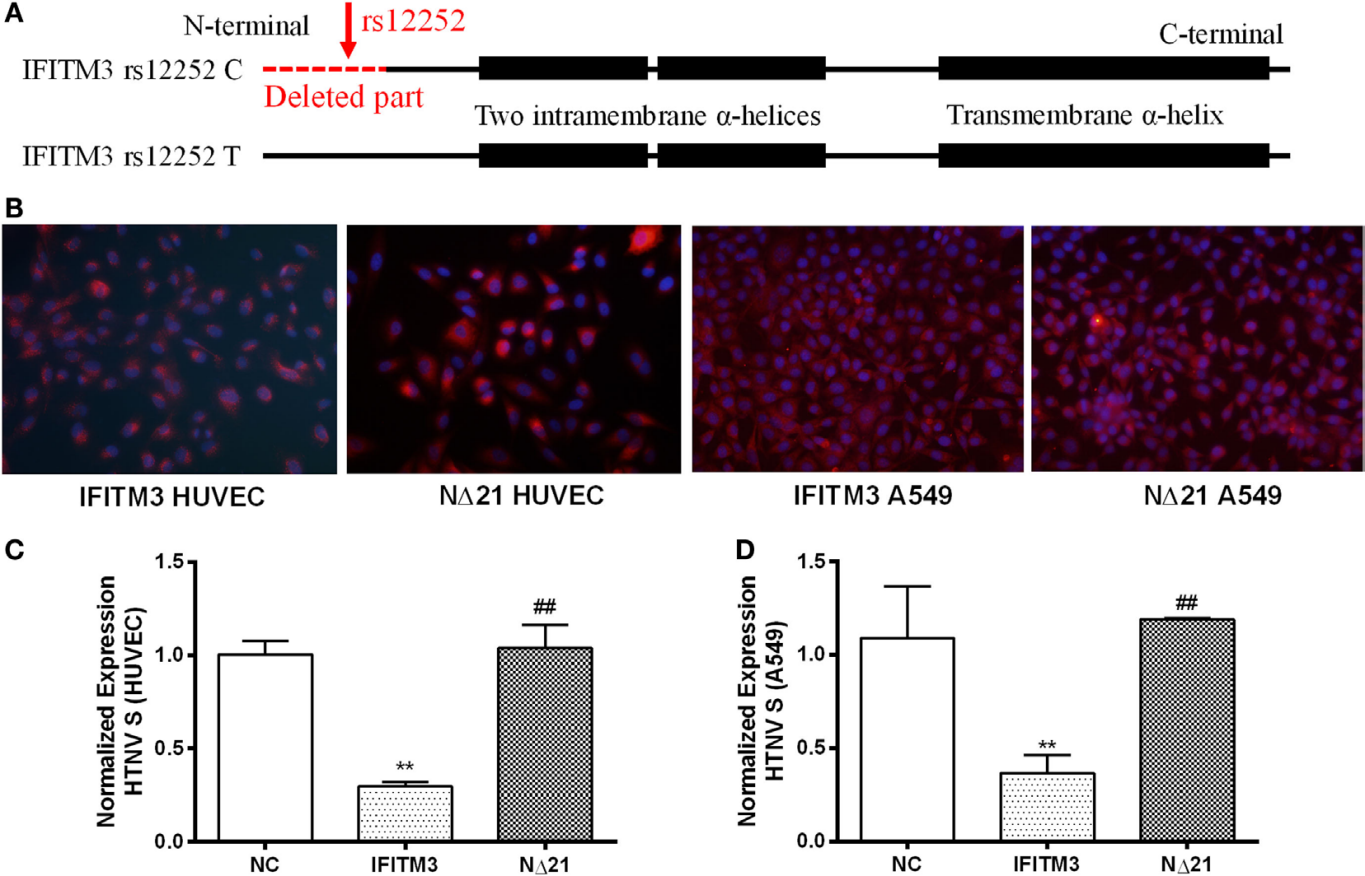
**The IFITM3 SNP rs12252 polymorphism influenced its anti-HTNV activity**. **(A)** The molecular dissection of IFITM3 protein and the location of SNP rs12252 (close to N-terminal showed by red arrow) which encodes a splice acceptor site altering T/C substitution mutation, resulting in a truncated protein with an N-terminal 21 amino acid deletion. The absence of the first N-terminal 21 amino acids was showed as red dotted line (deleted part). The C-terminal transmembrane α-helix and an N-terminal intramembrane segment was showed as black boxes. **(B)** HUVEC and A549 cells transduced to express either full-length (IFITM3) or truncated (NΔ21) IFITM3. c-myc-tag was stained red. **(C,D)** The expression of HTNV S segment in HUVEC and A549 cells transfected with empty vector (NC), full-length IFITM3, or truncated IFITM3 (NΔ21), determined by qPCR, *n* = 8. Data are presented as mean ± SEM. **P < 0.01 vs. NC, ^##^*P* < 0.01 vs. IFITM3.

Previous studies reveal that the truncated IFITM3 protein produced by SNP rs12252 C allele (Figure 2A, the missing part stands for the truncated 21 amino acids from N-terminal of IFITM3, the intramembrane helix, and transmembrane helix was presented as boxes) leads to an impaired anti-influenza activity (14). To test the functional significance of this polymorphism in HTNV infection, we transfected the majority T or minority C variant IFITM3 alleles that produce full-length or N-terminally truncated (NΔ21) proteins (Figure 2A) with c-myc-tag to HUVEC and A549 cell using lentivirus vectors (Figure 2B). Then, we challenged the cells with HTNV at moi = 1 for 24 h and found that cells with the minority C variant were more susceptible to HTNV infection with higher expression of HTNV S segment (Figure 2C) and more positive of HTNV NP (Figure S3 in Supplementary Material). Indeed, compared with the mock (empty vector)-infected control, the NΔ21 protein almost lost the ability to inhibit HTNV infection in both HUVEC and A549 cells (Figures 2C,D; Figure S3 in Supplementary Material).

### HTNV Infection Induced the Expression of IFITMs

**Figure 3:**
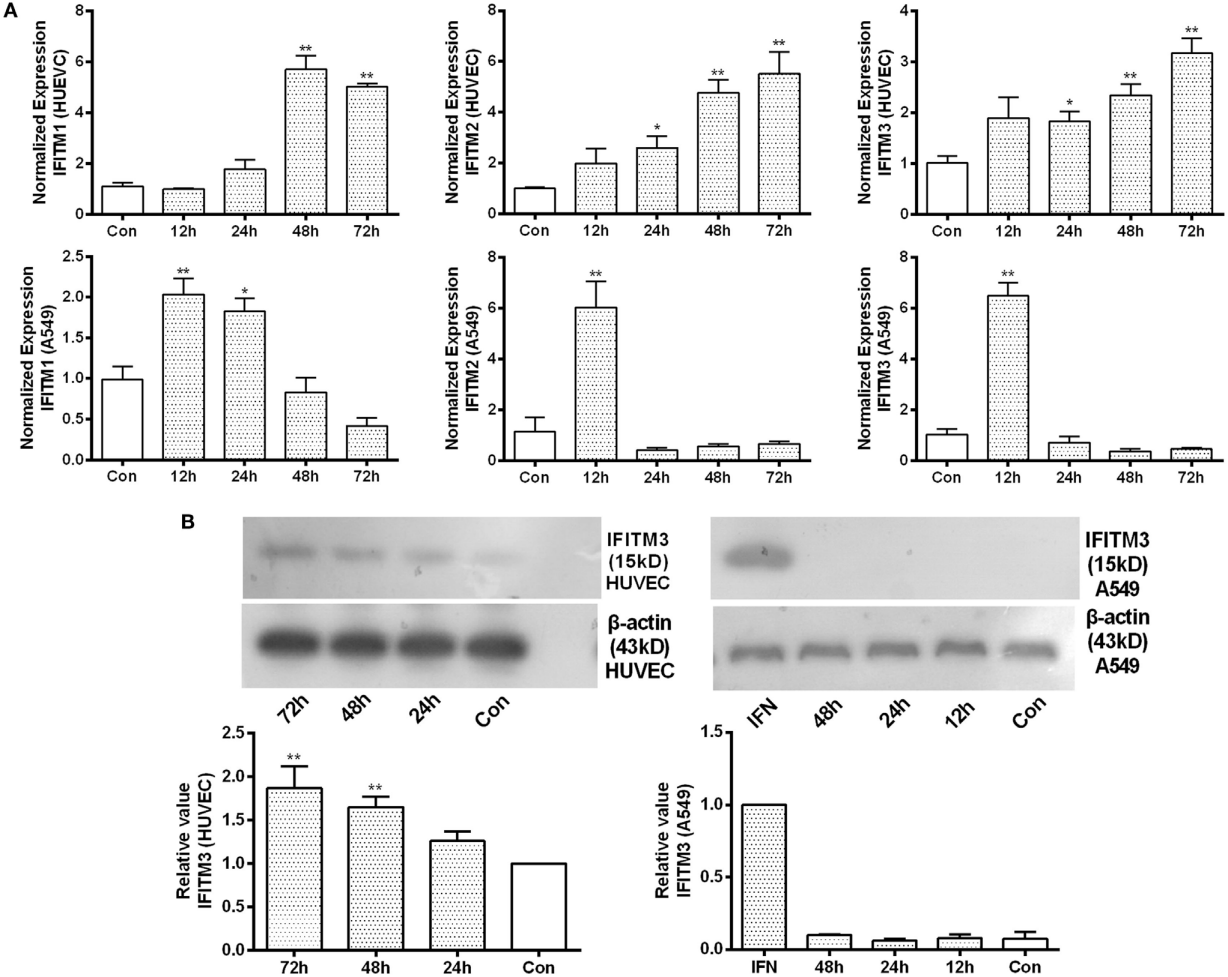
**Identification of IFITM3 alteration after HTNV infection**. **(A)** qPCR analysis of IFITM1, 2, and 3 in HUVEC and A549 cells infected by HTNV or Mock control (Con) at indicated time point post infection. **(B)** Western blot analysis of IFITM3 expression in HUVEC and A549 cells infected by HTNV or Mock control (Con) at indicated time point post infection. *n* = 8 for qPCR; *n* = 6 for Western blot analysis. Data are presented as mean ± SEM. Con: mock infection, 12–72 h: time postinfection, IFN: A549 cells treated with IFN-α2a (20 U/ml) serves as a positive control. *P < 0.05, **P < 0.01 vs. Con.

To determine the role of HTNV infection in inducing IFITMs, qPCR as well as Western blot of IFITMs were conducted in HUVEC and A549 cells (Figures 3A,B; Figure S1 in Supplementary Material). While we observed only a moderate upregulation of IFITM1, 2, and 3 mRNA and protein in HUVECs after more than 24 h postinfection; IFITM1, 2, and 3 mRNA, however, were only transiently upregulated in A549 cells and caused no significant change in protein level.

### Endogenous IFITM3 Contributed Significantly to the Anti-HTNV Effect of IFN-α2a

**Figure 4:**
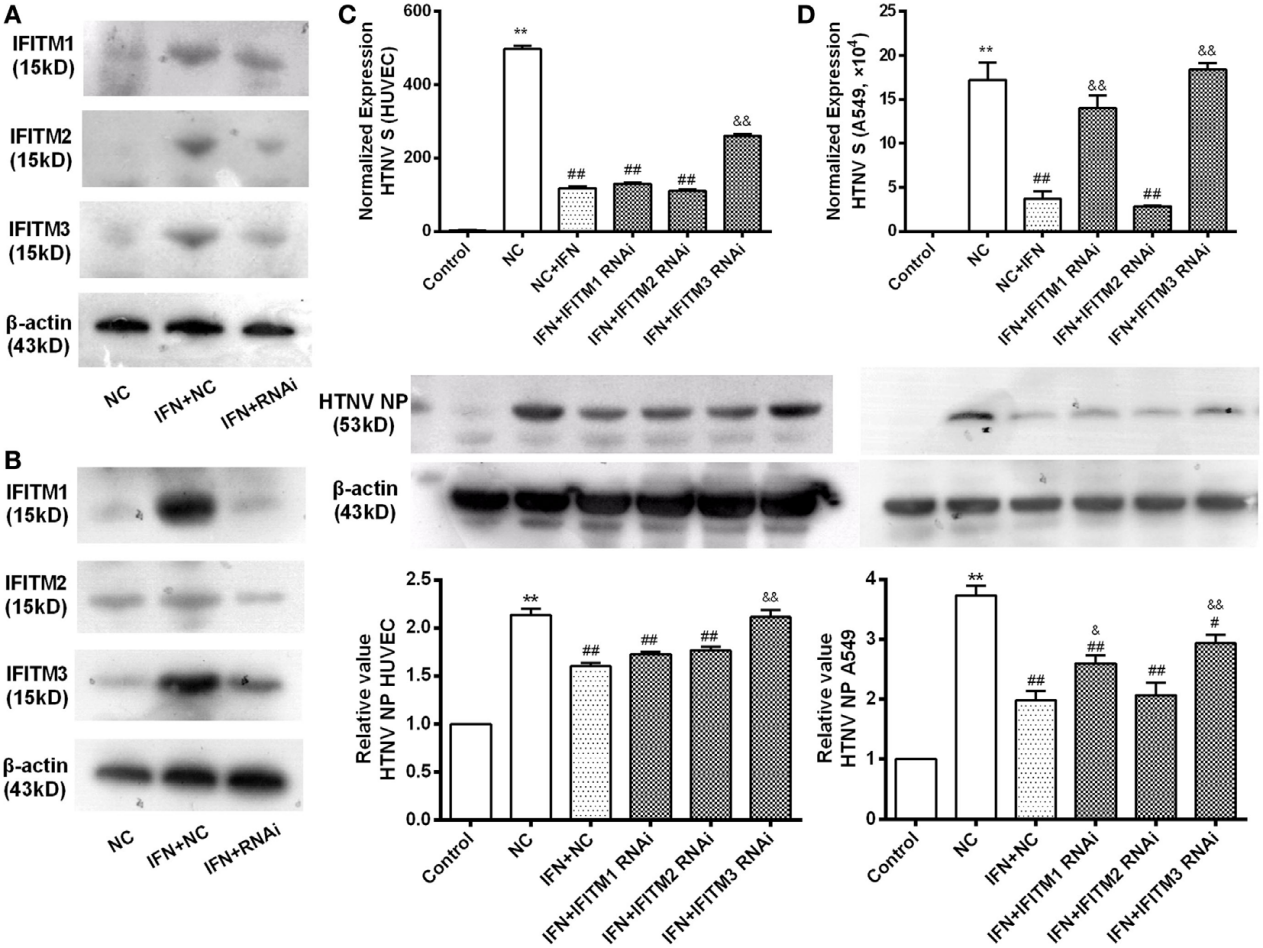
**Contribution of endogenous IFITMs to the anti-HTNV effect of IFN-α2a**. **(A,B)** Representative Western blot imaging for the effectiveness of the respective RNAi on IFITM1, 2, and 3 expression. A549 and HUVEC cells were transfected with indicated siRNAs for 12 h, treated with mock or IFN-α2a for 12 h, and then infected by HTNV (moi = 1) for another 24 h, followed by Western blot analysis, as described in Section “Materials and Methods.” **(C,D)** Effect of IFITM1, 2, and 3 RNAi on HTNV infection in HUVEC and A549 cells. The cells were treated as described above, HTNV replication was evaluated by qPCR for HTNV S segment (*n* = 8) and Western blot for HTNV NP (*n* = 6). Data are presented as mean ± SEM. Control: mock-infected and non-targeting siRNA group, NC: non-targeting siRNA group, IFN + NC: control siRNA + IFN-α2a group, IFN + RNAi:siRNA of the indicated IFITM + IFN-α2a group. **P < 0.01 vs. control, ^#^*P* < 0.05, ^##^*P* < 0.01 vs. NC, ^&^*P* < 0.05, ^&&^*P* < 0.01, vs. NC + IFN.

We knocked down the IFITM1, 2, and 3 expression by transfection of their siRNAs individually. The effect of siRNAs on the expression of target IFITMs was tested by qPCR in HUVECs (Figure S2 in Supplementary Material), and the effect of the best oligo against each IFITMs (IFITM1C, IFITM2A, IFITM3B) was tested by Western blot in A549 (Figure 4A) and HUVEC cells (Figure 4B). To assess the role of IFITMs in anti-HTNV effect of IFN-α2a, IFITM1, 2, and 3 were knocked down respectively by transfecting the above-tested oligoes for 12 h, followed by IFN-α2a treatment (20 IU/ml for another 12 h). The cells were then challenged with HTNV (moi = 1) for 24 h. The HTNV S segment and NP levels were significantly suppressed in both HUVEC and A549 cells in response to IFN-α2a treatment. Notably, knockdown of IFITM3 significantly restored the levels of HTNV S segment and NP in HUVEC and A549 cells. Knockdown of IFITM1 also partially restored the HTNV level in A549 cells (Figures 4C,D). These results demonstrate that endogenous IFITM3 significantly contributed to the anti-HTNV effect of IFN-α2a.

### Overexpression of IFITM3 Inhibited HTNV Infection by Preventing Cytoplasmic Entry

**Figure 5:**
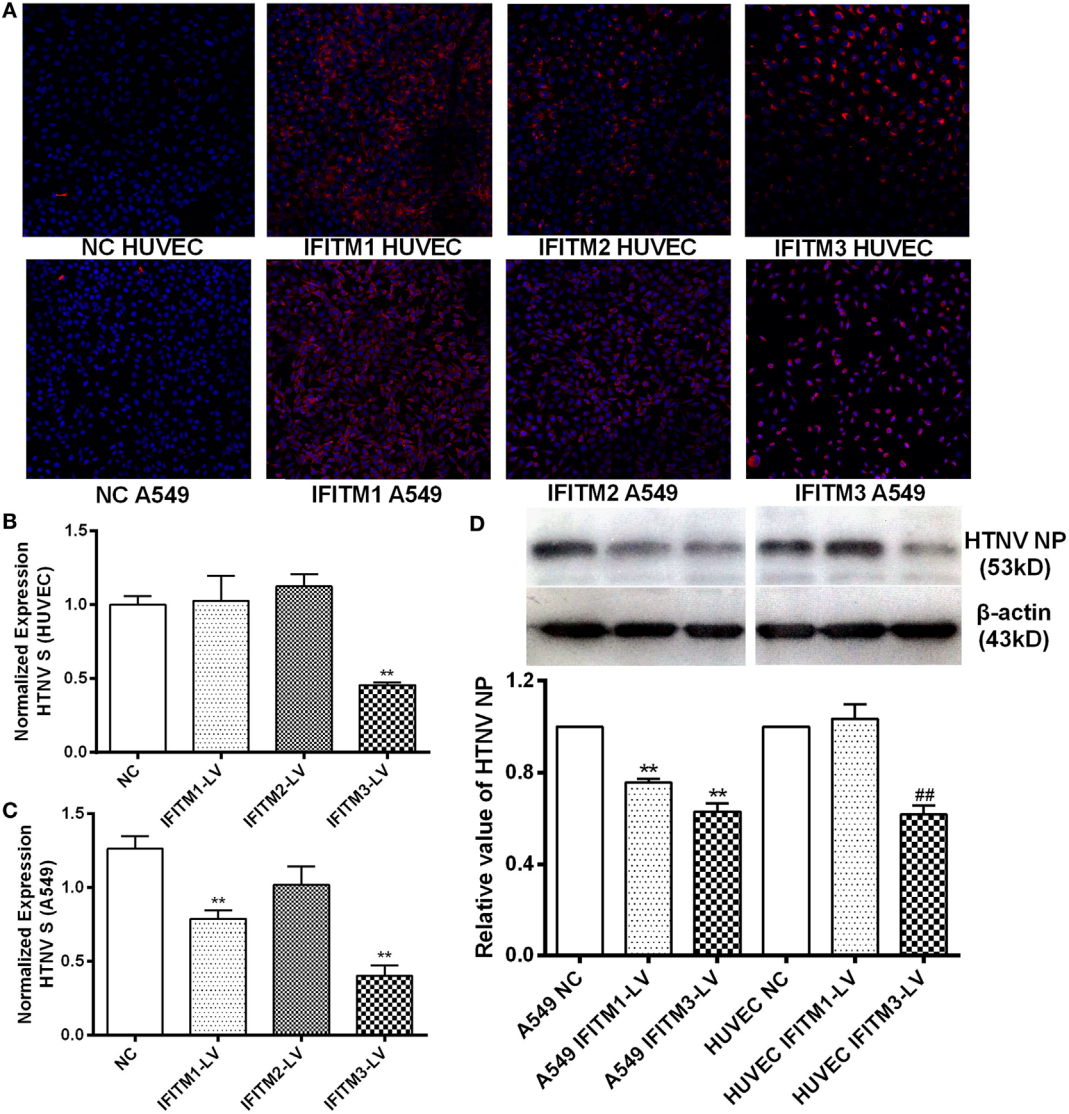
**Inhibitory effect of overexpressed IFITMs on HTNV infection**. **(A)** Confirmation of c-myc-tagged IFITM1, 2, and 3 overexpression by lentiviral vector. c-myc-tag was stained red, nucleus was counterstained with DAPI. **(B,C)** Effect of IFITM1, 2, and 3 overexpression on HTNV infection in HUVEC and A549 cells (moi = 1 for 24 h), assessed by qPCR for HTNV S segment. NC: control vector group, IFITM1-LV: IFITM1 overexpression group, IFITM2-LV: IFITM2 overexpression group, IFITM3-LV: IFITM3 overexpression group. Data are presented as mean ± SEM. *N* = 8. **P < 0.01 vs. NC **(D)** Effect of IFITM1, 2, and 3 overexpression on HTNV infection in HUVEC and A549 cells (moi = 1 for 24 h), tested by Western blot for HTNV NP. Data are presented as mean ± SEM. *N* = 6. **P < 0.01 vs. A549 NC in **(D)**, ^##^*P* < 0.01 vs. HUVEC NC.

**Figure 6:**
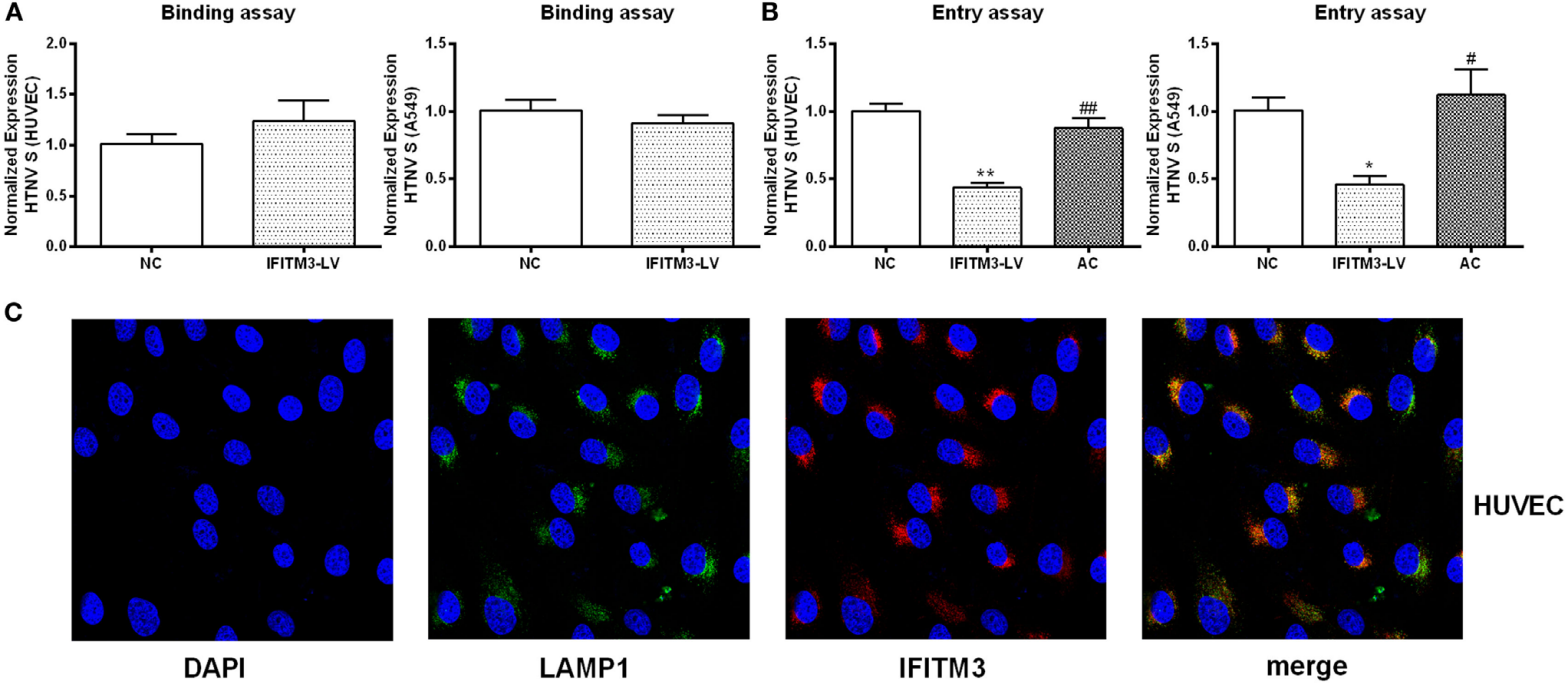
**IFITM3 inhibits HTNV entry into HUVEC and A549 cells and partially localizes to LAMP1^+^ late endosomes**. **(A,B)** The influence of IFITM3 on HTNV-HUVEC and HTNV-A549 cell binding and cellular entry. NC: control vector group, IFITM3-LV: IFITM3 overexpression group, AC: virus-plasma membrane fusion group, Data are presented as mean ± SEM, *n* = 8. *P < 0.05, **P < 0.01 vs. NC, ^#^*P* < 0.05, ^##^*P* < 0.01 vs. IFITM3-LV. **(C)** IFITM3 partially localizes in LAMP1^+^ late endosome in HUVEC. Nucleus is counterstained with DAPI. IFITM3 (red) and LAMP1 (green) co-localization (yellow and orange dots) is evaluated by confocal microscopy.

To assess the anti-HTNV effects of IFITMs, we tested the effect of overexpressed IFITM1, 2, and 3 on HTNV infection. c-myc-tagged IFITM1, 2, and 3 were expressed in both HUVEC and A549 cells (Figure 5A), and the cells were then challenged with HTNV (moi = 1) for 24 h. The HTNV S segment and NP levels were suppressed by IFITM3 overexpression in HUVEC cells (Figures 5B–D). They were also suppressed by expressing IFITM1 and IFITM3 in A549 cells (Figures 5B–D). The inhibitory effect of IFITM3 was further confirmed by immunofluorescence analysis of HTNV NP (Figure S3 in Supplementary Material). These results were in accordance with the above-described RNAi results. To determine whether IFITM3 inhibited HTNV binding or entry, HUVEC and A549 cells were incubated with HTNV (moi = 1) at 4°C for 1 h, unbound virus was washed away, and HTNV RNA collected at this time point represents HTNV bound to the cell surface. After virus binding, the cells were shifted to 37°C for 2 h to allow HTNV internalization, and HTNV RNA collected at this time point represents cell-internalized virus. As a positive control for inhibition of virus entry, we incubated a parallel group of cells with HTNV at pH = 5.5 as described in Section “Materials and Methods.” Expression of IFITM3 did not affect HTNV binding (Figure 6A) but significantly suppressed HTNV entry in both HUVEC and A549 cells (Figure 6B).

### IFITM3 Was Partially Localized to LAMP1^+^ Late Endosomes in the Host Cells

To elucidate the mechanism of IFITM3 function, we investigated the subcellular localization of IFTIM3 in the host cells. IFITM3 was found partially localized to LAMP1^+^ late endosomes in HUVECs analyzed by confocal microscopy (Figure 6C). The co-localization of IFITM3 and LAMP1^+^ late endosomes had also been found in A549 cells (27). Because the transfer into LAMP1^+^ late endosomes is a necessary step for HTNV entry (28), this result provides an evidence for the anti-HTNV mechanism of IFITM3.

### LncRNA NRIR Negatively Regulated the Initial Transcription of IFITM3 and Facilitated HTNV Infection

**Figure 7:**
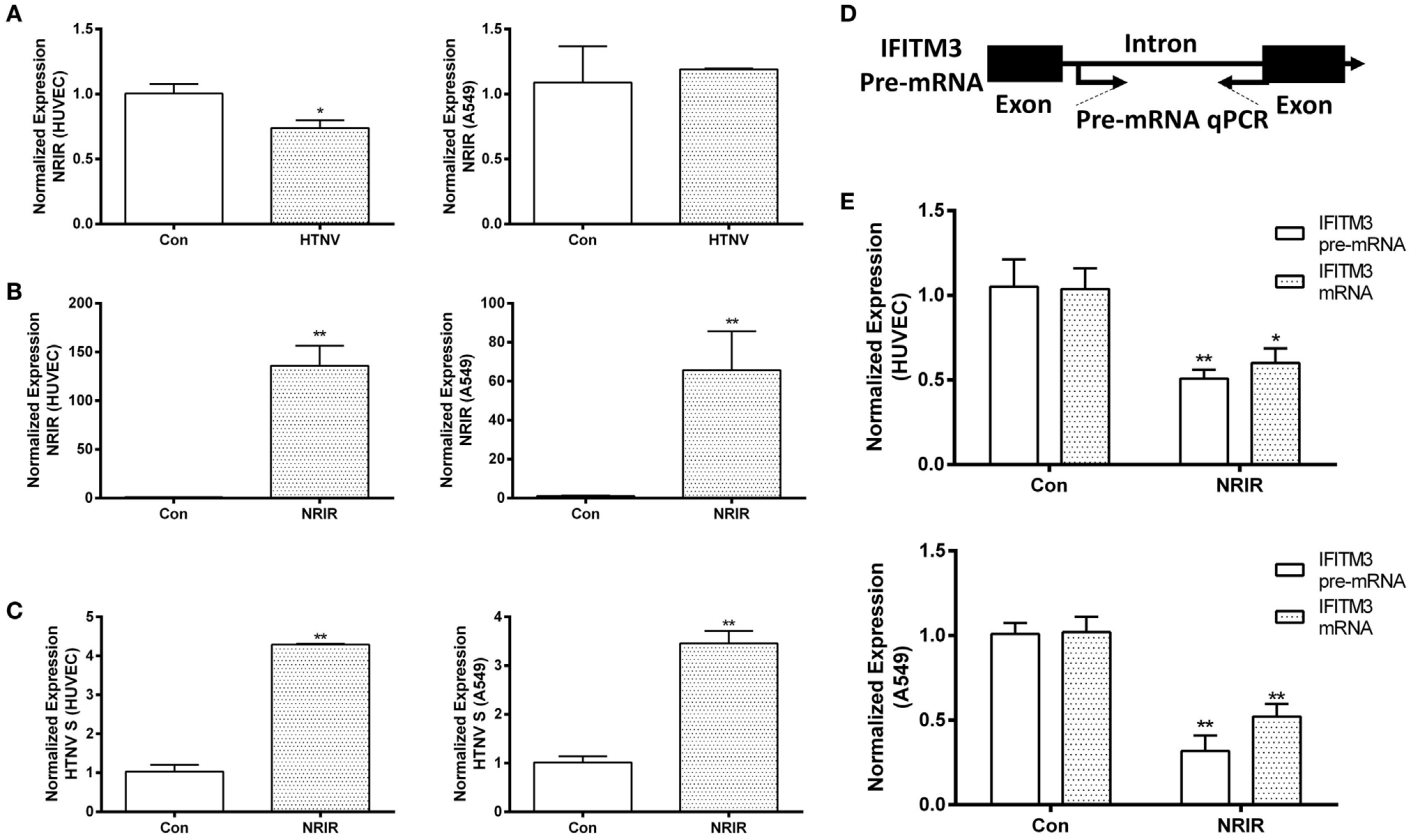
**NRIR negatively regulates the initial transcription of IFITM3 and facilitates HTNV infection**. **(A)** qPCR analysis of NRIR downregulation in HUVEC, but not in A549 cells, infected by mock control (Con) or HTNV, 48 h postinfection. **(B)** Overexpression of NRIR in HUVEC and A549 cells transfected by mock control (Con) or NRIR expressing plasmid, confirmed by qPCR. Values are mean ± SEM, *n* = 8, *P < 0.05, **P < 0.01 vs. Con. **(D)** Schematic dissection of IFITM3 pre-mRNA exons and intron for designing primers of qPCR analysis of IFITM3 pre-mRNA levels. **(C,E)** NRIR suppresses IFITM3 transcription in HUVEC and A549 cells and facilitated HTNV infection. Con: empty vector control, NRIR: NRIR overexpression group. Values are mean ± SEM, *n* = 8, *P < 0.05 vs. Con, **P < 0.01 vs. Con.

LncRNA- and microRNA-mediated regulation of IFITM3 has been reported in several studies. We tested the change of previously reported regulators of IFITMs, such as NRAV, NRIR, and miR-130a after HTNV infection, among which NRIR was the only changed one (downregulated) after HTNV infection (Figure 7A; Figure S4 in Supplementary Material) in HUVEC. However, the expression of NRIR was unchanged in A549 cells. We overexpressed NRIR in HUVEC and A549 cells using the pcDNA3.1 vector (Figure 7B). Importantly, overexpression of NRIR significantly suppressed IFITM3 mRNA and pre-mRNA levels and facilitated HTNV infection in HUVEC and A549 cells (Figures 7C–E). These data suggest that lncRNA NRIR is a negative regulator of IFITM3 transcription.

## Discussion

Hantaan virus is an enveloped, negative-sense RNA virus from the genus Hantavirus within the family Bunyaviridae. It causes HFRS, which is an important threat to public health worldwide. It is also a potential weapon for biological terrorism. Reservoir animals, usually rodents, are asymptomatic during persistent infection. Unlike in rodents, Hantavirus infection leads to HFRS and Hantavirus pulmonary syndrome (HPS) in humans (21). The major clinical characteristics of HFRS include fever, hemorrhage, hypotension, and renal injury (1, 21), causing severe manifestations and death in some cases. The current standard of care for HFRS relies on symptomatic and supportive treatment. It has been confirmed that the plasma viral load is associated with the severity of HFRS, implicating the importance of viremia in the pathogenesis of HFRS (2). However, no direct antiviral medications are currently available for this illness. Interferon is the key molecule for the antiviral response and has been used as an antiviral medicine in many diseases. It has been reported that HTNV infection induces a late type I interferon response (16). However, the set of ISGs required for IFN-mediated inhibition of HTNV has not yet been identified. Therefore, identification of ISGs that are effective against HTNV is an attractive strategy to identify novel therapeutic targets.

In this study, we demonstrated a significantly high frequency of the rs12252 C allele and CC genotype among HFRS patients with severe illness compared with mildly infected individuals and the healthy Han Chinese. The rs12252 C allele and CC genotype are also found to be associated with higher plasma viral load in the early stage of HFRS. We also discovered that HTNV infection induces IFITMs, and the truncated IFITM3 produced by rs12252 C allele exhibits significantly decreased anti-HTNV activity. Interestingly, IFITM3 is found to restrict HTNV infection with a mechanism of cellular entry inhibition. Indeed, IFITM3 is localized to the late endosome in the host cells, which is a necessary structure for HTNV entry. In addition, we find that HTNV infection downregulated lncRNA NRIR 48 h post infection, which negatively regulates the transcription of IFITM3. Collectively, these results suggest that IFITM3, regulated by NRIR, inhibits HTNV infection, and its SNP rs12252 correlates with the disease severity and viral load in patients with HFRS.

The antiviral properties of IFITM proteins were identified in 2009 in an RNAi screen for host factors that influence influenza virus replication (29). IFITM1, 2, and 3 have been demonstrated to possess antiviral activity in several studies. Everitt et al. demonstrated that the severity of influenza virus infection was greatly increased in IFITM3-knockout mice compared with wild-type animals (15). Different IFITM members have also been confirmed to inhibit the cellular entry of multiple virus families (including *filoviruses, rhabdoviruses*, and *flaviviruses*) (7, 9–11, 30). For example, HIV-1 and HCV infection are inhibited by IFITM1 (31–34). It is commonly believed that IFITMs restrict viral infection at the stage of cellular entry (12). Recent studies suggested that the cellular location of different IFITMs may influence the range of viruses restricted by each protein (5). IFITM1 prevents HCV entry because it colocalizes with CD81 on the cell membrane, interrupting the endocytosis of HCV particles (32), whereas IFITM3 confines influenza virus in acidified endosomal compartments (27). Notably, retrovirus subvirus particles (ISVPs), which do not require endosomal acidification for entry, are not inhibited by IFITM3 expression, suggesting that IFITM3 may function at the stage of endosomal entry (35). Studies utilizing cell–cell fusion assays have suggested that IFITM3 blocks the entry of enveloped virus by preventing the fusion of the viral membrane with a limiting membrane of the host cell, either the plasma membrane and/or the endosomal membranes. The results obtained using two-photon laser scanning and fluorescence lifetime imaging (FLIM) suggest that IFITM proteins may reduce membrane fluidity and increase the spontaneous positive curvature in the outer leaflet of membranes (36). In the present study, we demonstrated that IFN-α2a (20 U/ml) significantly inhibited HTNV infection, siRNA-mediated depletion of IFITM3 alone significantly mitigated the antiviral effect of IFN-α2a in both HUVEC and A549 cells, whereas depletion of IFITM1 alone alleviated the antiviral effect of IFN-α2a in A549 cells. Overexpression of IFITM3 inhibited HTNV infection to HUVEC and A549 cells. IFITM1 overexpression was also effective in inhibition of HTNV in A549 cells. All these results suggest that IFITM3 is an important control factor under natural infection of HTNV. Our results also demonstrate that the effectiveness of IFITM3 is cell type-independent, which is in accordance with the results from similar viruses, such as RVFV (18). Binding and entry assays, conducted by controlling the temperature and pH, showed that IFITM3 did not significantly influence HTNV binding but inhibited HTNV entry into HUVEC and A549 cells. Indeed, IFITM3 partially localizes to the late endosome of the host cells, which is a necessary site for the HTNV entry. However, we failed in tracking the transportation of HTNV in infected cells possibly due to the lack of fluorescence-labeled virus. In addition, IFITM1 also suppressed HTNV infection in A549 cells. The mechanism underlying anti-HTNV effect of IFITM1 remains undetermined and deserves to be further explored.

According to a recent study on the three-dimensional structure of IFITM3, there is a C-terminal transmembrane α-helix and a two-N-terminal intramembrane α-helices (shown in Figure 2A as black boxes) in IFITM3 (14). There are two splice variants that differ by the presence or absence of the first N-terminal 21 amino acids (deleted part, shown in Figure 2A as red dotted line). Several SNPs including 13 non-synonymous, 13 synonymous, 1 in-frame stop, and 1 splice site acceptor-altering have been reported in the translated IFITM3 sequence (15, 29). Among them, the rare SNP rs12252C allele of IFITM3 truncates the protein as described above, leading to a reduced inhibition of influenza virus infection in A549 cells (15). We demonstrated that truncated IFITM3 protein also loses the ability to inhibit HTNV infection *in vitro*. In Northern European patients hospitalized with seasonal influenza or pandemic influenza A virus, increased homozygosity of the minor C allele of SNP rs12252 in IFITM3 was observed (37). In Chinese patients infected with influenza A (H1N1) virus, there was also an increased frequency of the C allele and CC genotype of SNP rs12252 (13). In the present study, we observed an increased frequency of the C allele and CC genotype of SNP rs12252 in severely infected HFRS patients compared with healthy control and mildly affected patients. Patients carrying the CC genotype also had higher plasma viral loads compared with those with the CT/TT genotype. Given the impaired function of the IFITM3 protein produced by the C mutation, and the fact that enrichment of the rs12252 C allele in patients with severe disease and the higher viral load in patients with the CC genotype, this founding suggests that IFITM3 plays a pivotal role in the anti-HTNV response *in vivo*. We speculate that the much higher level of CC allele at healthy population of Han Chinese compared with Caucasians may place the Chinese at a higher risk for developing severe illness upon HTNV infection, which needs further investigation.

LncRNAs are a group of non-coding RNAs longer than 200 nt that function as gene regulators, playing a role in regulating multiple cellular functions, including the innate immunity. For example, lncRNA NEAT1 is reported to be upregulated by influenza virus or PolyI:C stimulation, which promotes IL-8 expression (38). lncRNA NRAV has been shown to negatively regulate the initial transcription of IFITM3 and Mx1 by affecting the histone modification of these genes (25). lncRNA NRIR is a non-coding ISG, which has been reported to negatively regulate IFITM1 and Mx1 expression in HCV infection (20). Mir-130a was also reported as a regulator of IFITM1 (23). In this analysis, lncRNA NRIR was downregulated in HUVECs after HTNV infection for 48 h, overexpression of NRIR negatively regulates the initial transcription of IFITM3, evidenced by the decreased pre-mRNA as well as mRNA levels. NRIR overexpression also facilitated HTNV infection. These results indicate that the downregulation of NRIR after HTNV infection is possibly involved in the activation of innate immune responses against HTNV infection. We have also evaluated other potential regulators of IFITM3 before we choose NRIR for further study. Another lncRNA that can regulate IFITM3, i.e., NRAV (NR_038854), remained unchanged after HTNV infection (Figures S4A,B in Supplementary Material). Additionally, miR-130a, which potentially regulate IFITM3, was also unaltered after HTNV infection (Figures S4C,D in Supplementary Material).

In conclusion, this study revealed a critical role for IFITM3 in HTNV infection. We demonstrated, for the first time to our knowledge, that IFITM3 is a newly identified anti-HTNV ISG; its expression is negatively regulated by NRIR; and its antiviral activity seems *via* a mechanism of inhibiting virus entry into the host cells. In addition, we discovered that the IFITM3 SNP rs12252 C allele and CC genotype correlates with the plasma HTNV load and the severity of HFRS; and the rs12252 C allele produces a truncated IFITM3 protein (NΔ21) that attenuates its anti-HTNV function. These results provide new insights into the role of IFITM3 in regulating innate immunity against HTNV infection, which is the basis for identifying new targets to develop novel agent against this worldwide infectious disease.

## Author Contributions

ZX-y, BP-y, YC-t, and MH-w performed the experiments; WP-z, BX-f, LY-f, ZY, and JZ-s designed the research; HC-x, YW, and WX analyzed the data; TK and ZC-m provided clinical data; ZX-y and BP-y wrote the paper.

## Conflict of Interest Statement

The authors declare that this research was conducted in the absence of any commercial or financial relationships that could be construed as a potential conflict of interest.
