# Supplementary material for: Interferon-Induced Transmembrane Protein 3 Inhibits Hantaan Virus Infection, and Its Single Nucleotide Polymorphism rs12252 Influences the Severity of Hemorrhagic Fever with Renal Syndrome
Source: Front Immunol. 2017 Jan 3;7:535. doi: 10.3389/fimmu.2016.00535 (PMC5206578; doi:10.3389/fimmu.2016.00535)
Supplement: Supplementary file 1 [file Data_Sheet_1.DOCX]

Supplementary Material

**Interferon Induced Transmembrane Protein 3 Inhibits Hantaan virus Infection and its Single Nucleotide Polymorphism rs12252 Influences the Severity of Hemorrhagic Fever with Renal Syndrome**

Zheng Xu-yang^1+^, Bian Pei-yu^1+^, Ye Chuan-tao^1^, Ye Wei^2^, Ma Hong-wei^2^, Tang Kang^3^, Zhang Chun-mei^3^, Lei Ying-feng^2^, Wei Xin^1^, Wang Ping-zhong^1^, Huang Chang-xing^1^, Bai Xue-fan^1^, Zhang Ying^1*^, Jia Zhan-sheng^1*^

*** Correspondence:** Dr. Jia Zhan-sheng: [jiazsh@fmmu.edu.cn](mailto:jiazsh@fmmu.edu.cn) Or Dr. Zhang Ying: zyfmmu@hotmail.com

# Supplementary Figures and Tables

## Supplementary Figures


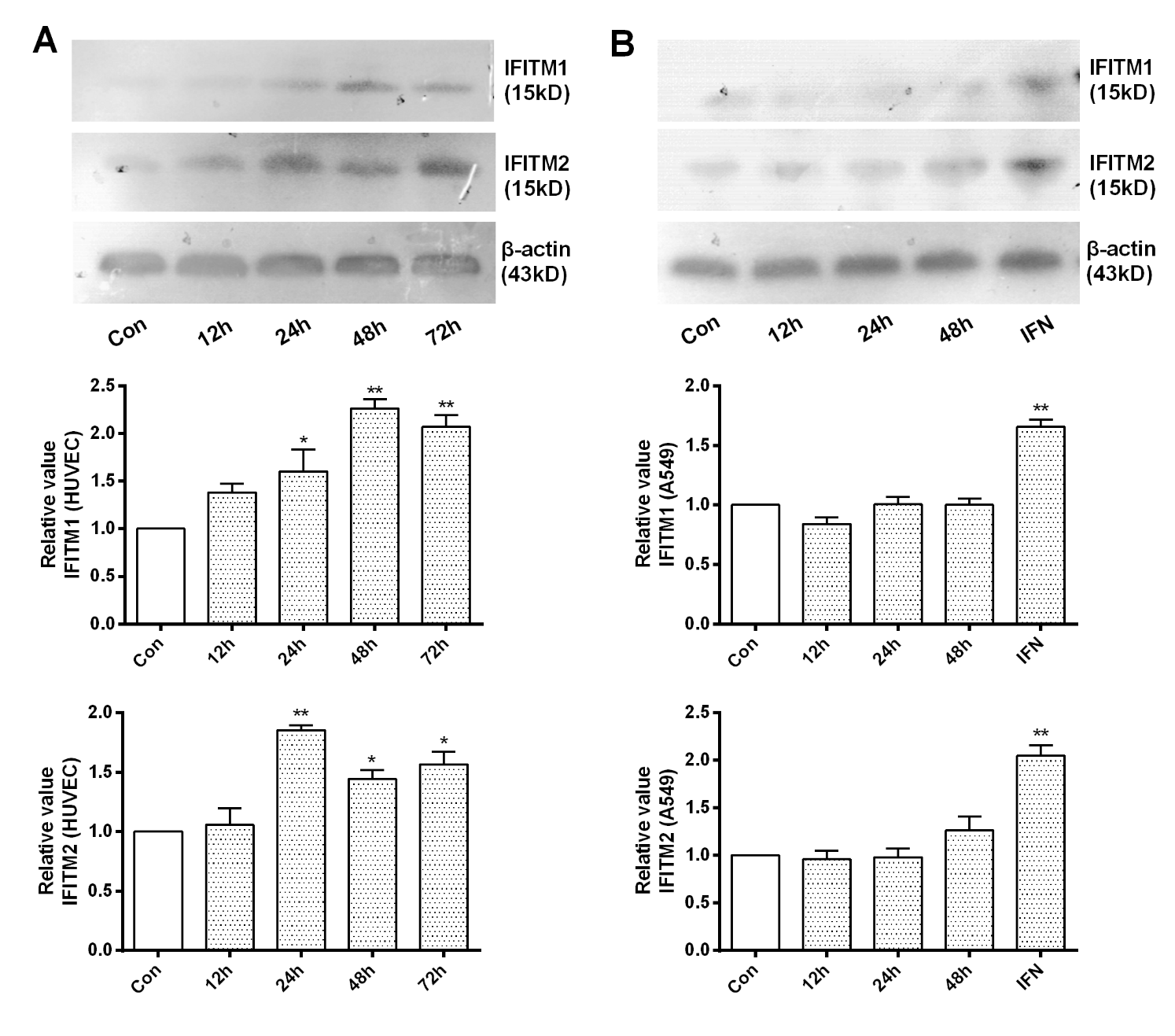


**Supplementary Figure 1.** Western-blot analysis of the IFITM1,2 expression after HTNV infection (moi=1). HTNV infection induced up-regulation of IFITM1, 2 in HUVEC (A, B) but not in A549 (A, C) cells. n=6 in Western-blot. Data are expressed as mean±SEM. Con: Mock infection, 12h-72h: time post infection, IFN: cells treated with IFN-α2a (20U/ml) as positive control. ^*^*P*<0.05, ^**^*P*<0.01 vs. Con.


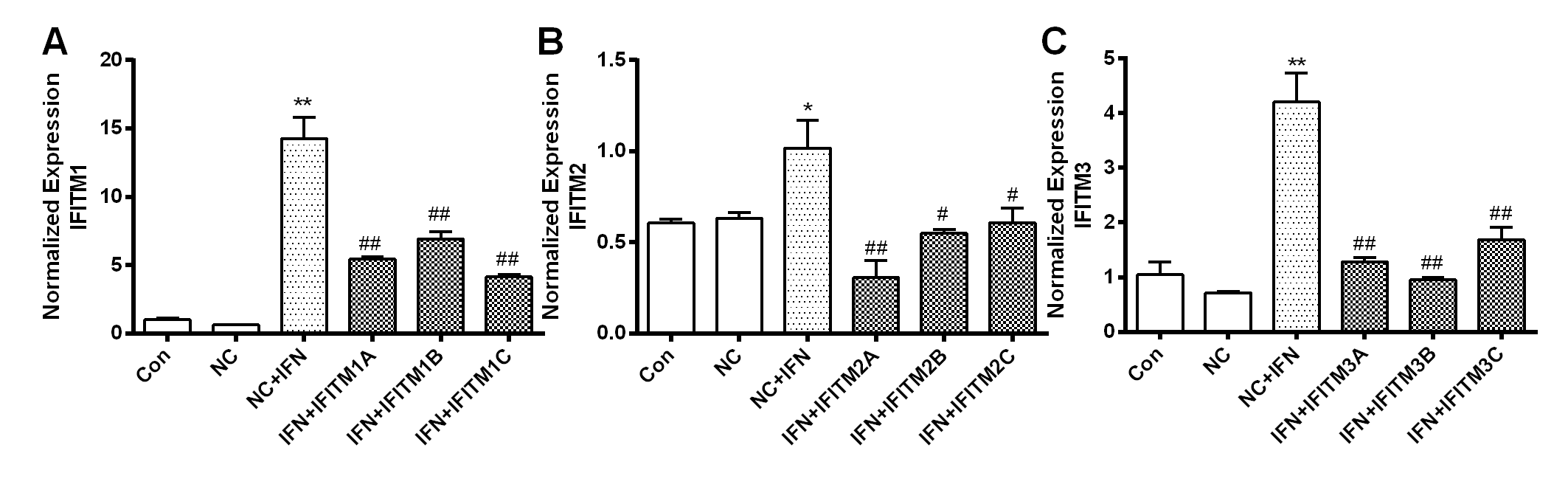


**Supplementary Figure 2.** Test of the efficiency of siRNAs targeting IFITM1,2,3 by qPCR in HUVEC. Test of the efficiency of siRNAs targeting IFITM1 (A), IFITM2 (B), IFITM3 (C). A, B, C in each figure stand for 3 different siRNAs provided by Origene for indicated IFITM gene. We chose IFITM1C, IFITM2A and IFITM3B to perform Western-blot since these siRNAs had best silencing performance. n=6. Data are expressed as mean±SEM. ^*^*P*<0.05, ^**^*P*<0.01 vs. NC, ^#^*P*<0.05, ^##^*P*<0.01 vs. NC+IFN.

**
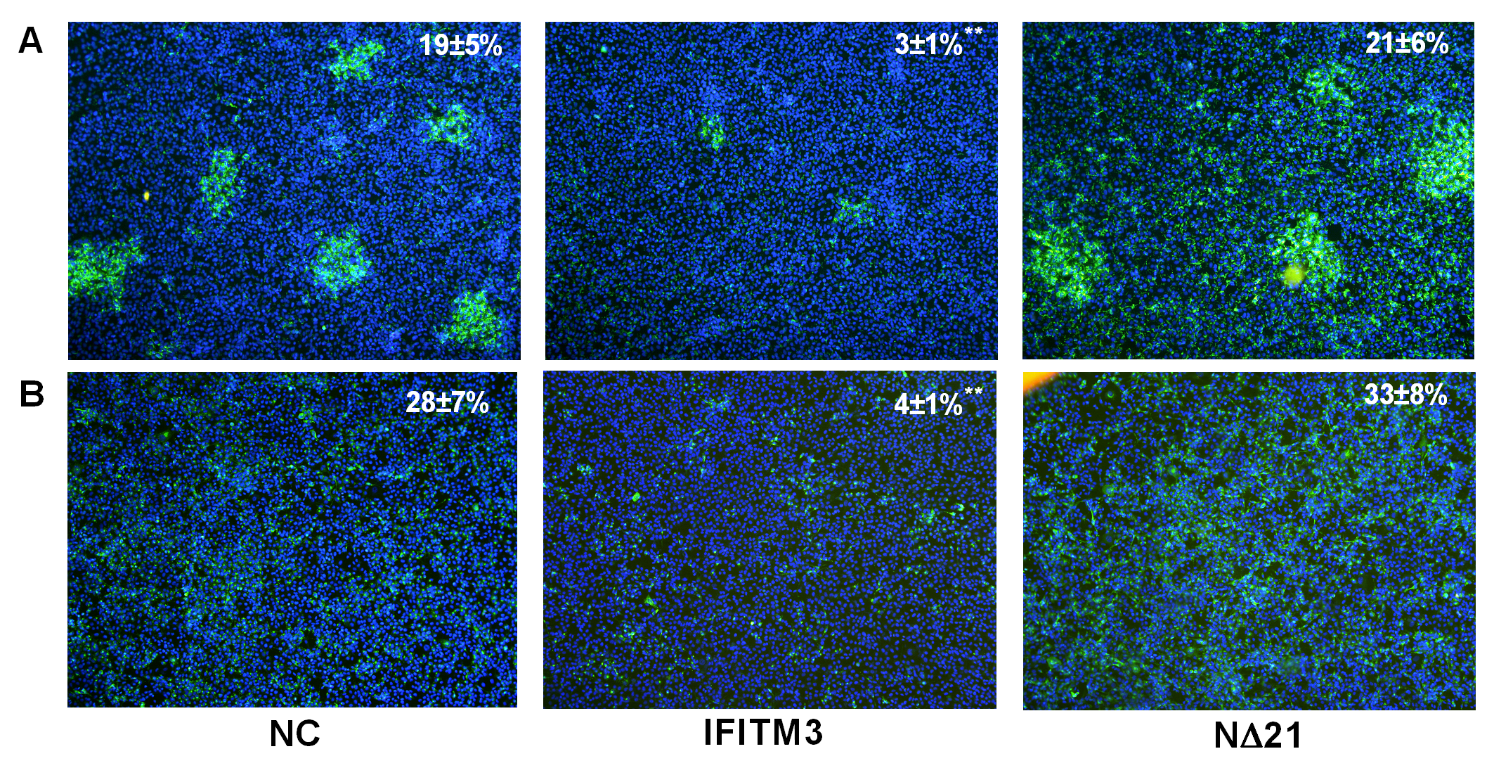
**

**Supplementary Figure 3.** Overexpression of IFITM3 but not NΔ21 inhibits HTNV infection tested by Immunofluorescence assay. HTNV NP positive cells were detected by immunofluorescence (green) in both HUVEC (A) and A549 cells (B). n=5. The percentage of HTNV NP positive cells different groups, expressed by mean±SEM, are shown. ^**^*P*<0.01 vs. NC.


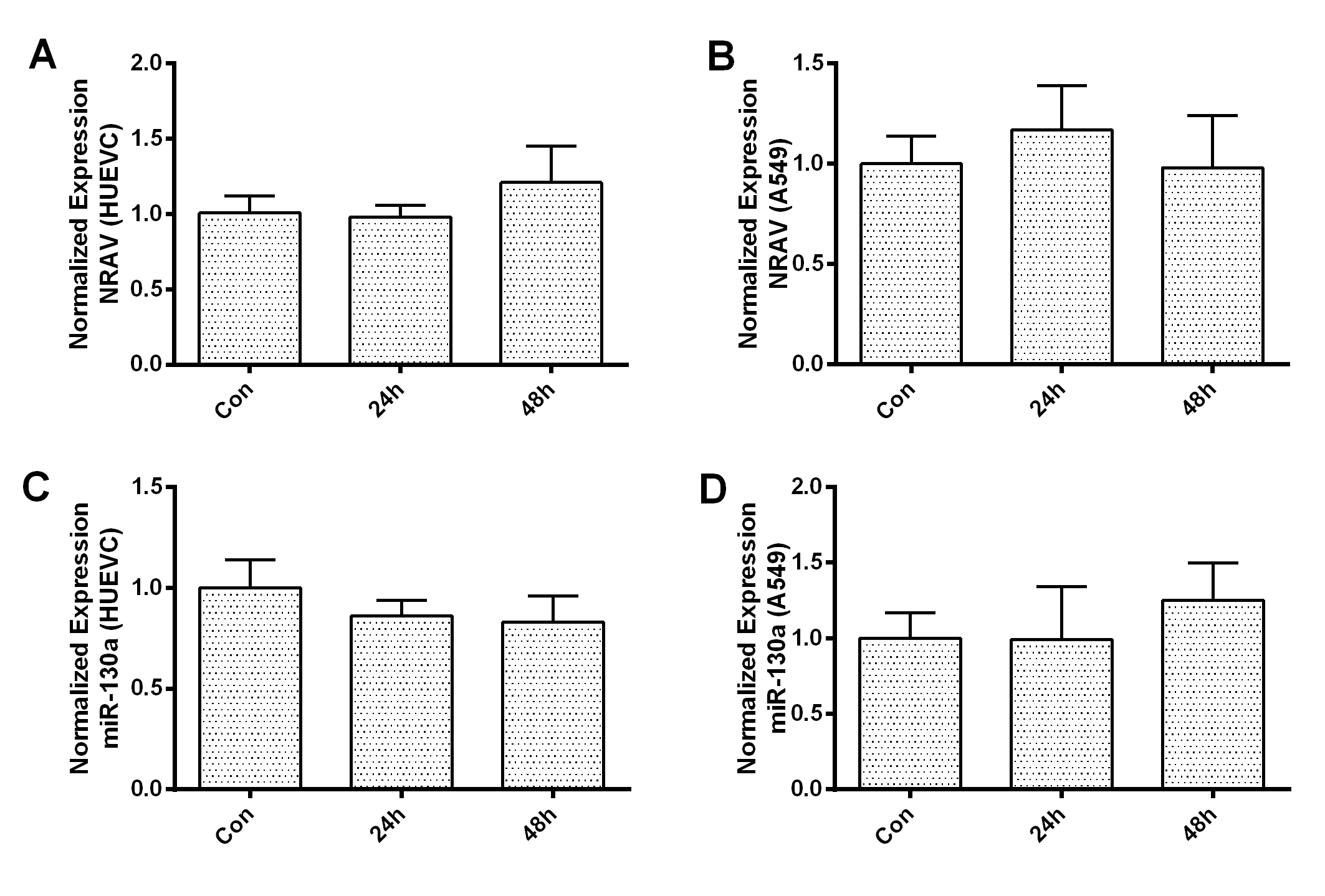


**Supplementary Figure 4.** The expression of other potential regulators of IFITM3 after HTNV infection (moi=1). LncRNA NRAV remained unchanged after HTNV infection in HUVEC and A549 cells (A, B). Mir-130a also remained unaltered after HTNV infection in HUVEC and A549 cells (C, D). Con: Mock infection, 24h-48h: time post infection
